# Using a novel cellular platform to optimize CRISPR/CAS9 technology for the gene therapy of AIDS

**DOI:** 10.1007/s13238-017-0453-z

**Published:** 2017-08-18

**Authors:** Jingjin He, Thanutra Zhang, Xuemei Fu

**Affiliations:** 1 The Eighth Affiliated Hospital of Sun Yat-sen University, Shenzhen, 518033 China; 2 Division of Biological Sciences, University of California, San Diego, 9500 Gilman Drive, La Jolla, CA 92093 USA; 3 Shenzhen Children’s Hospital, Shenzhen, 518026 China

**Dear Editor,**

Despite tremendous effort devoted to the development of antiretroviral therapies to combat HIV over the past decades, AIDS remains one of the most important global infectious diseases. According to UNAIDS report on the global AIDS epidemic in 2016, the estimated number of people living with HIV rose from 7.5 million in 2010 to 36.7 million in 2015. Furthermore, drug-resistance HIV strains have recently been reported (Wensing et al., 2017). Therefore, it is important to develop new therapies to eliminate HIV in the patients. Immortalized cell lines representing the major targets of HIV in human are important for HIV research and therapeutic development. In this context, HIV mainly targets macrophage and CD4^+^ T lymphocytes *in vivo* (Iordanskiy et al., 2013). In addition, the co-receptors CCR5 and CXCR4 are required for the HIV infection of T cells (Moore et al., 1997; Zaitseva et al., 1998). Therefore, immortalized CD4^+^ T cell stably expressing HIV-1 co-receptor CCR5 or CXCR4 will be highly useful for HIV research. However, most of the T cell lines generally do not express adequate level of CCR5 to support the infection of CCR5 tropic HIV-1. Even if many previously established T cell lines could overcome this restriction after transduction of the expression vectors (Wu et al., 2002; Krowicka et al., 2008), the random integration in these lines leads to the unstable expression of transgene and also could affect the expression of the nearby genes (Modlich et al., 2005; Nienhuis et al., 2006).

Recently, CRISPR/CAS9 technology has become a powerful tool for efficient gene editing by inducing DNA double-strand breaks at the designated gene locus to stimulate gene mutation or homologous recombination (Hsu et al., 2014). Due to its high specificity and low off-target mutation rate (Sander and Joung, 2014; Veres et al., 2014), CRISPR/CAS9 technology is powerful for gene editing in human cells. Therefore, we employed CRISPR/CAS9 technology to develop a novel CCR5-expressing CD4^+^ T cell line by inducing the expression of CCR5 from its endogenous locus by inserting the CAG promoter into the promoter of the CCR5 gene via homologous recombination. The resulting cell line is permissive for HIV infection and is useful for developing gene therapy of AIDs.

We used Jurkat T cell line as the parental cell line because it already expresses high levels of endogenous CD4 and CXCR4 genes. To induce the expression of the endogenous CCR5 gene in Jurkat T cells, we knock-in the ubiquitous and strong promoter, cytomegalovirus enhancer fused to the chicken beta-actin promoter (CAG), into the promoter of CCR5 gene. The knock-in strategy is described in Fig. 1. The guide RNA was used to target and introduce the double strand break (DSB) at the designated locus as indicated by asterisk in Fig. 1A. The schematic diagram of the wild type and knock-in alleles is shown (Fig. 1A and 1B). Homologous recombination events were screened by PCR (Fig. 1C). The homologous recombination events were confirmed by Southern blotting, further indicating the lack of any random integration (Fig. 1D). The resulting knock-in cell line is denoted Jurkat-KI-R5. Flow cytometric analysis of Jurkat-KI-R5 cells shows high expression levels of surface CCR5 in Jurkat-KI-R5 cells, indicating that the knock-in strategy works as expected to induce the expression of the endogenous CCR5 (Fig. 1E).

**Figure 1:**
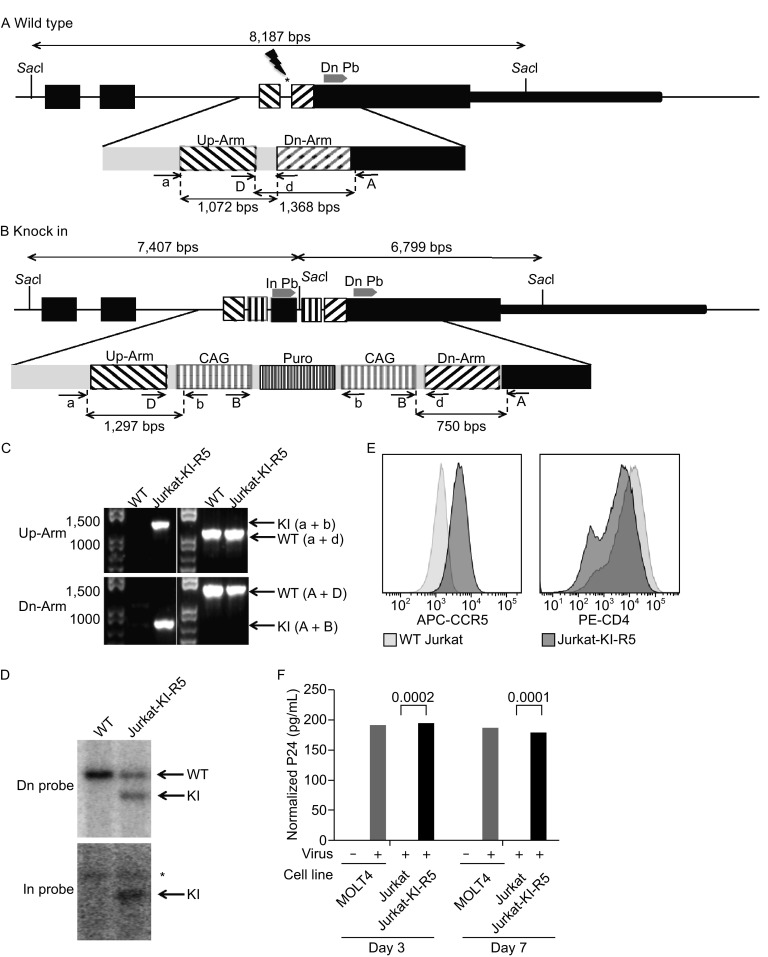
**Knock-in of the ubiquitous CAG promoter into the promoter region of the endogenous** ***CCR5*** **gene of Jurkat T cell line**. (A) The configuration of the endogenous human CCR5 locus. The black box represents the exon of CCR5. Two homologous arms are indicated by Up-Arm and Dn-Arm. The asterisk indicates the targeting site of gRNA. (B) The configuration of knock-in allele. The location of internal and downstream probes for Southern blotting and the sizes of *Sac*I restriction fragments are shown. PCR primers (a, d, A, and D) were used to identify wild type allele. Primers (a, b, A, and B) were used to screen knock-in clone. Primers a and b are specific for identifying upstream arm recombination and primers A and B are specific for downstream arm recombination. Size of PCR amplicon is shown below each primer set. (C) PCR analysis of CAG knock-in clone. Genomic DNA isolated from individual clones was examined by PCR. Primers a and b amplify a 1,297-bp fragment, and primers A and B amplify a 750-bp fragment from knock-in allele. (D) Knock-in recombination event was confirmed by Southern blotting analysis. Genomic DNA was digested with *Sac*I and hybridized to the downstream (Dn) and internal (In) probes, respectively. The wild type (WT) and knock-in (KI) bands are indicated. Asterisks indicate bands generated by single-crossover at the downstream homologous arm. (E) Surface CCR5 is highly expressed in CD4^+^ Jurkat-KI-R5. (F) Jurkat-KI-R5 permits the infection of HIV-1. Three and seven days after HIV infection, supernatants were collected and analyzed for HIV-1 p24 antigen. HIV-1 entry was only detected in Jurkat-KI-R5 but not in the parental Jurkat cells

The main purpose to generate Jurkat-KI-R5 cells is to create a permissive cell line for HIV infection. Therefore, we examined the susceptibility of Jurkat-KI-R5 cell to the infection of CCR5 tropic HIV. In contrast to the Jurkat cells that produce no detectable p24 antigens in the supernatant after HIV, high levels of p24 antigen were detected in the supernatant of Jurkat-KI-R5 cells after 3 days and 7 days of HIV infection (Fig. 1F). Therefore, Jurkat-KI-R5 cells represent a much-needed cellular platform to support future HIV research.

To further demonstrate the feasibility to use Jurkat-KI-R5 cells for HIV research, we employed the Jurkat-KI-R5 cells to develop gene therapy for AIDS. The highly pursued strategy for gene therapy of HIV infection is to disrupt CCR5 expression in CD4^+^ T cells. This will generate CD4^+^ T cells that are resistant to HIV infection and help to eliminate the infected cells. A pair of guide RNA was designed to target the CCR5 exon before the ∆32 exon, a natural 32-bp deletion mutation leading to resistance to HIV infection in humans (Fig. 2A) (Samson et al., 1996). We cloned the paired gRNAs into the plasmid expressing either Cas9 or Cas9n, the D10A mutant nickase version of Cas9 that mediates genome editing with improved specificity (Ran et al., 2013). We also synthesized the chemically modified sgRNAs and CAS9 mRNA, which are more stable and shown to significantly improve genome-editing efficiency after directly transfecting into cells (Hendel et al., 2015). Seven days after transfecting the plasmid or chemically modified sgRNA/Cas9n mRNA into the Jurkat-KI-R5 cells, the disruption of the CCR5 gene was evaluated by flow cytometry. FACS analysis demonstrated that the CCR5 gene is disrupted in over 50% of Jurkat-KI-R5 cells with either Cas9 or Cas9n, and cells transfected with sgRNA/Cas9n mRNA achieved nearly 80% of knockout efficiency (Fig. 2B). Efficient CCR5 disruption in Jurkat-KI-R5 cells with either Cas9 or Cas9n plasmid was further confirmed by Western blotting analysis of the CCR5 protein and by PCR assay (Fig. 2C and 2D).

**Figure 2:**
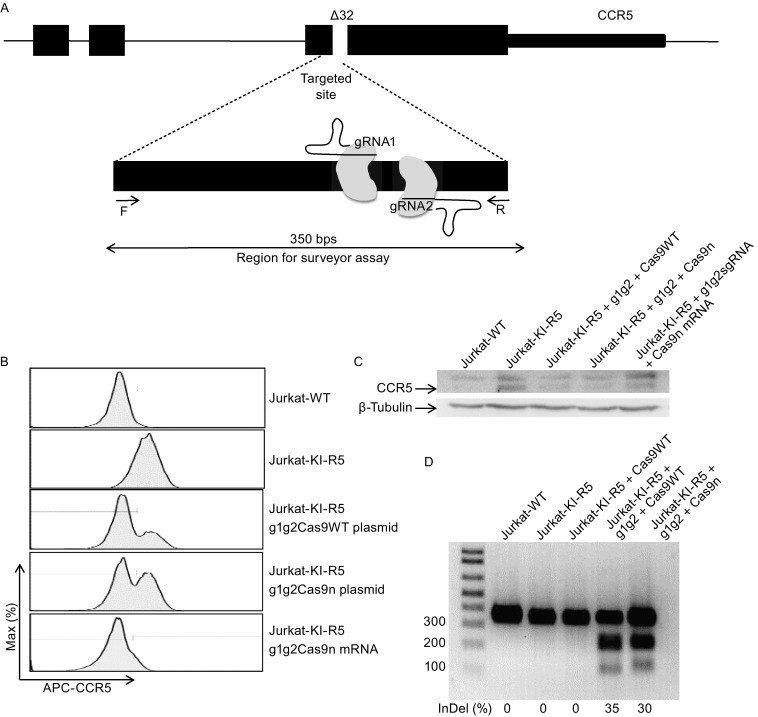
**Efficient disruption of CCR5 in Jurkat-KI-R5 cells using plasmid or RNA-based CRISPR/CAS9 technology**. (A) Schematic diagram of the dual gRNAs targeting the endogenous CCR5 gene. Forward and reverse primers were used to amplify the region for T7 Endonuclease I assay. (B) Loss of CCR5 surface expression in Jurkat-KI-R5 cells seven days after the transfection of plasmid expressing Cas9/Cas9n and gRNAs or chemically modified sgRNA/Cas9n mRNA. (C) CCR5 protein was overexpressed in Jurkat-KI-R5 cells and silenced seven days after transfecting either the plasmid expressing Cas9/Cas9n and gRNAs or chemically modified sgRNA/Cas9n mRNA. CCR5 and loading control β-actin are indicated with arrowheads. (D) Efficiency of CCR5 disruption in Jurkat-KI-R5 transfected with plasmid expressing Cas9/Cas9n and gRNA1, 2 was assessed by surveyor assay. Percentage of gene disruption is indicated under each panel

One of the key bottlenecks for HIV research is the lack of HIV permissive human cell lines. Taking advantage of the CRISPR/CAS9 gene editing technology, we generated a human T cell line that stably expresses CD4 and both co-receptors CCR5 and CXCR4. This T cell line is different from previously reported CCR5 transgenic T cell line because the CCR5 gene is expressed from its endogenous locus. The feasibility to use Jurkat-KI-R5 cells for HIV research is further supported by the findings that these cells are highly susceptible for HIV infection. Jurkat-KI-R5 cells can be useful in many aspects of HIV research and therapy development, such as HIV drug resistance, efficacy of new antiretroviral therapy, and gene therapy. In support of this notion, we used the Jurkat-KI-R5 cells to evaluate the efficiency of CCR5 disruption with CRISPR/CAS9 gene editing technology. Our data demonstrate that both CRISPR/Cas9 and CRISPR/Cas9n can efficiently ablate CCR5 with paired gRNAs in T cell lines. In this context, it remains difficult to disrupt CCR5 gene in the primary human CD4^+^ T cells with single gRNA (Mandal et al., 2014). As reported previously (Ran et al., 2013), paired nicking can reduce off-target activity by 50 to 1,500-fold in cell line. This could improve the safety of this technology for clinical application. The chemically synthesized and stabilized gRNA/mRNA can further increase the efficiency of CCR5 disruption. Considering that CRISPR/CAS9 gene editing technology could induce off-target genomic mutations, the Jurkat-KI-R5 cells can be further employed to improve the efficiency and safety of CRISPR/Cas9 mediated disruption of the CCR5 gene.

## Electronic supplementary material

Below is the link to the electronic supplementary material.

Supplementary material 1 (PDF 56 kb)
