# Supplementary material for: Using a novel cellular platform to optimize CRISPR/CAS9 technology for the gene therapy of AIDS
Source: Protein Cell. 2017 Aug 18;8(11):848–52. doi: 10.1007/s13238-017-0453-z (PMC5676591; doi:10.1007/s13238-017-0453-z)
Supplement: Supplementary file 1 — Supplementary material 1 (PDF 56 kb) [file 13238_2017_453_MOESM1_ESM.pdf]

SUPPLEMENTARY DATA

Methods

Cell culture

Jurkat cells were maintained at a cell density of 0.5-2*10^6 cells/ml in RPMI 1640 supplemented with 10% heat-inactivated fetal bovine serum, 100 units/ml penicillin, 100 μg/ml streptomycin and 2 mmol/L L-glutamine.

DNA vector construction

The vector encoding human Cas9 was obtained from Addgene. The vector producing gRNA to target CCR5 was constructed from phU6-gRNA plasmid (Addgene) by ligating the annealed oligonucleotides of gRNA into a unique BbsI site. The targeting site is GTGCACAGGGTGGAACAAGA. The donor plasmid providing repair template included Loxp-flanked selection cassette pCAG/Neo/IRES/polyA, two CCR5 homology arms and CAG promoter. Upstream homology arm was cloned in front of selection cassette. CAG promoter and downstream homology arm were inserted after selection cassette. The construction of donor plasmid was accomplished using Gibson's method and verified by sequencing analysis.

Jurkat cell transfection and selection

Jurkat cells were co-transfected with linearized donor plasmid, pU6-CCR5gRNA and pCAG-hCas9 using the Gene Pulser II cell electroporation system. Cells were recovered and selected by culturing in growth medium containing puromycin at the concentration of 1.75 μg/mL. After 7 days, survival cells were seeded in 96-well plate to isolate the single clone. In gene therapy study, Jurkat cells were transfected with a Cas9 (Cas9n) and paired gRNAs encoding plasmid, or

sgRNAs chemically modified at both termini with 2'-O-methyl-3'-phosphorathioate in combination with Cas9n mRNA using Amaxa Nuclefector kits.

Flow cytometric analysis

The flow cytometric analysis of the surface expression of human CCR5 and CD4 was performed by BD LSR-II machine. The data were further analyzed by using FACS Diva (Becton Dickinson) and FlowJo software. Specifically, $1x10^6$ cells were washed with PBS and stained with DAPI, APC anti-hCCR5 (BD PharMingen) and PE anti-CD4 (BD PharMingen) for 30 minutes at room temperature. Then cells were washed with PBS twice and re-suspended with FACS buffer.

PCR analysis

PCR was performed to identify the homologous recombinants to insert the selection cassette and CAG promoter in CCR5 locus. Forward primer a (5'-tctatgaccttccctgggactt-3') and reverse primer d (5'-cttgttccaccctgtgcataa-3') were used to identify wild type allele at the upstream part of targeting site. Forward primer A(5'-tttcgacaccgaagcagagt-3') and reverse primer D(5'-gtgcatgttctttgtgggct-3') were used for downstream part. To identify knock-in recombination, same forward primers but different reverse primers were used. Reverse primer b (5'-tcgaacgtaaactcctcttcagac-3') was used to validate recombination at the upstream arm, while reverse primer B (5'-cgtgctggttattgtgctgtc-3') were specific to downstream recombination.

Southern blot analysis

Genomic DNA from Jurkat and Jurkat-CCR5 were extracted and 15 ug were digested with SacI. The digested DNA was separated on a 1.0% agarose gel, transferred to nitrocellulose, and probed for puromycin resistance and CCR5 gene using a ^{32}P-labeled DNA fragment obtained by PCR amplification.

HIV infection assay

Jurkat and Jurkat-KIR5 were infected with BaL HIV-1 at 0.3MOI for 2 hours to initiate an infection. MOLT-4 with infection was the positive control and MOLT-4 without infection was negative control. Cell free supernatant was collected at day 3 and 7 after the infection and tested for P24 level. All test conditions were done in triplicate. Statistical analysis was done by Prism6 program.

T7 Endonuclease I Assay

Genomic DNA was extracted from modified and control cells. Amplicons spaning the targeted regions were PCR amplified using Q5 polymerase. T7 endonuclease I (NEB) was used according to the manufacturer's instruction.
